# Hydroxypropyl-*β*-Cyclodextrin Complexes of Styryllactones Enhance the Anti-Tumor Effect in SW1116 Cell Line

**DOI:** 10.3389/fphar.2020.00484

**Published:** 2020-04-22

**Authors:** Ru Ma, Jie-tao Chen, Xiao-yue Ji, Xiao-li Xu, Qing Mu

**Affiliations:** ^1^School of Pharmacy, Fudan University, Shanghai, China; ^2^School of Chemistry and Chemical Engineering, Queen's University Belfast, Belfast, United Kingdom; ^3^Cancer Hospital, Fudan University, Shanghai, China

**Keywords:** styryllactone, absolute configuration, HP-*β*-CD, apoptosis, DSC

## Abstract

Styryllactones, a class of compounds obtained from the genus *Goniothalamus* (Annonaceae), have demonstrated *in vitro* antitumor activity. However, the aqueous solubility of these compounds is poor. In this study, we identified the absolute configurations of the previously isolated compounds, which were first isolated in our laboratory, by single-crystal X-ray diffraction analysis using Cu Kα radiation. Subsequently, the antitumor activities of the compounds were evaluated by 3-(4,5-dimethylthiazol-2-yl)-2,5-diphenyl-tetrazolium bromide staining in four tumor cell lines. The induced apoptosis activity of leiocarpin E-7ʹ-Monoacetate was studied by an annexin V fluorescein isothiocyanate/propidium iodide double-staining experiment, and the caspase activity was tested in the SW1116 cell line. The results demonstrated that the antitumor activities of cheliensisin A and goniodiol-7-monoacetate were limited by their poor water solubility. To address this issue, hydroxypropyl-*β*-cyclodextrin (HP-*β*-CD) complexes of the compounds were synthesized by the saturated aqueous method. The complexes were then analyzed using a differential scanning calorimeter. The IC_50_ of cheliensisin A was reduced by 45% and 58% against SW1116 and SMMC-7721 cell lines, respectively. Similarly, the IC_50_ of goniodiol-7-monoacetate was reduced by 55% and 34% against the two tumor cell lines, respectively. To further evaluate whether the styryllactones and complexes possessed selectivity against cancer cell lines and normal cell lines, toxicity against human normal cell line (HEK293T) was evaluated. The results demonstrated that the HP-*β*-CD complexes displayed more cytotoxicity than the respective pristine compounds against the HEK293T cell line. However, there existed a therapeutic window when the complexes were applied against cancer cell lines. In summary, the synthesis of several styryllactone compounds complexed with HP-*β*-CD was reported for the first time. These complexes could significantly enhance the cytotoxic effects of styryllactone compounds.

## Introduction

Colon cancer is the third most common type of cancer worldwide in both men and women, and is associated with a high recurrence rate and increasing mortality rate (Lao and Grady, 2011; Purushotham et al., 2012; Altobelli et al., 2014; Siegel et al., 2014; Sunkara and Hebert, 2015). The existing treatment regimens for colon cancer include chemotherapy, radiotherapy, and surgical ablation. Among these, chemotherapy is the most common strategy (Wang et al., 2014b; Pohl and Schmiegel, 2016). However, the two major challenges for the effective treatment of colon cancer are adverse effects due to cancer chemotherapy and drug resistance (Kozovska et al., 2014; Lim et al., 2019). Hence, it is imperative to search for new chemotherapeutic agents that have better safety and efficacy profiles. In this context, the application of natural compounds is a promising approach (Sridhar et al., 2014; Levrier et al., 2015; Wang et al., 2016).

**Figure 1:**
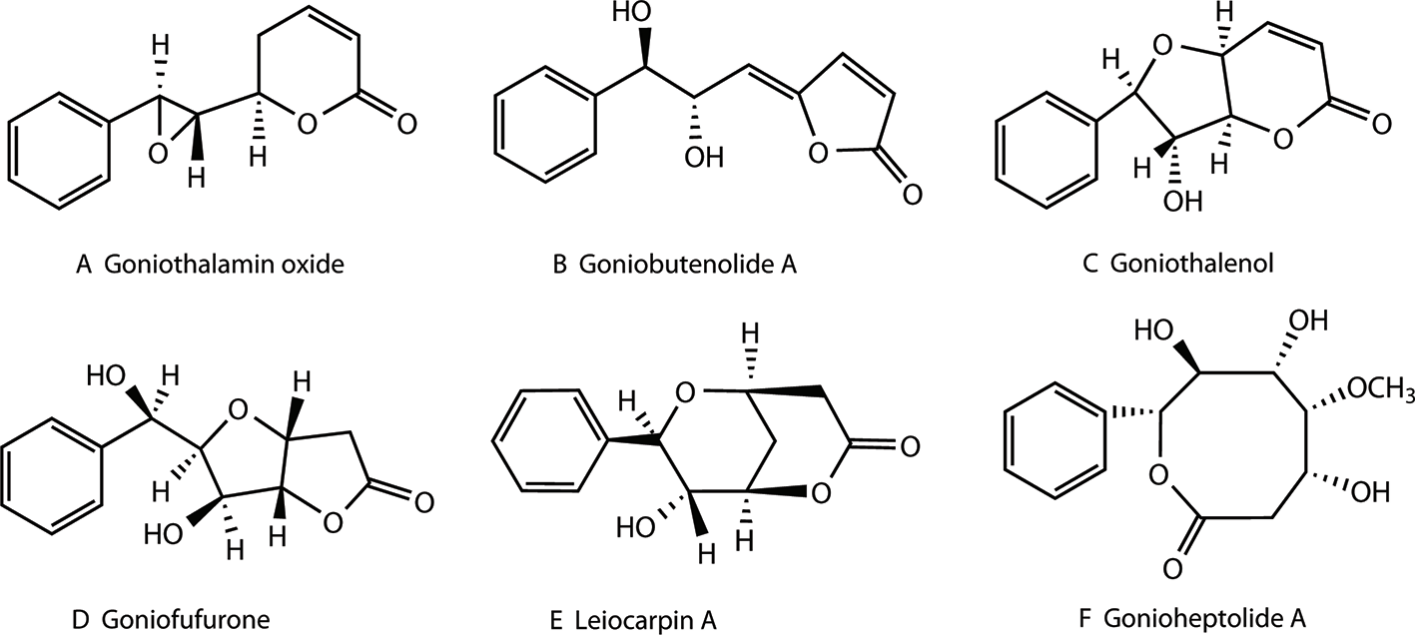
Structures of different styryllactones. Styryllactones are classified based on different structural skeletons as follows: styrene -pyrone **(A)**, styrene -furanone **(B)**, furan -pyrone **(C)**, furan -furanone **(D)**, pyran -pyrone **(E)**, and heptyl esters **(F)**.

Styryllactones represent a series of natural products, isolated exclusively from the genus *Goniothalamus* belonging to the Annonaceae family, which are mostly indigenous in southeast Asia. Styryllactones are classified based on different structural skeletons as follows (Bermejo et al., 1997; Bermejo et al., 1998; Cao et al., 1998; Bermejo et al., 1999; Hu et al., 1999; Mu et al., 1999a; Peris et al., 2000; Lan et al., 2005; Liou et al., 2014): styrene–pyrone (**Figure 1A**), styrene–furanone (**Figure 1B**), furan–pyrone (**Figure 1C**), furan–furanone (**Figure 1D**), pyran–pyrone (**Figure 1E**), and heptyl esters (**Figure 1F**).

Studies have shown that some styryllactones possess potent cytotoxicity against human colon tumor cell lines. Ali et al. reported that goniothalamin exhibited the highest cytotoxic activity against HGC-27 cells among the different cell lines tested (HGC-27, MCF-7, PANC-1, HeLa) (Ali et al., 1997). Vendramini-Costa et al. demonstrated the importance of goniothalamin as a proapoptotic, and therapeutic agent for the treatment inflammatory bowel disease and emphasized its potential as a chemopreventive agent for colon cancer (Vendramini-Costa et al., 2016). Cheliensisin A, a novel styryllactone isolated from *Goniothalamus cheliensis* Hu, could trigger p53-mediated apoptosis, accompanied by dramatic inhibition of the anchorage-independent growth of HCT116 cells, thus highlighting its potential cancer therapeutic effect (Zhang et al., 2014).

In recent years, mechanisms related to the antitumor activity of styryllactone compounds have been reported. For example, goniothalamin induced the release of inflammatory cytokines by upregulating the B-cell lymphoma-2 (Bcl-2)-associated X protein (Bax)/Bcl-2, phosphorylate c-Jun N-terminal kinase (p-JNK1)/JNK1, and p-p38/p38 ratios, which led to cleavage of poly (ADP-ribose) polymerase (PARP) and, finally resulted in apoptosis of the HT-29 cells (Vendramini-Costa et al., 2016). The cells were unable to grow without the BIRC 5 (Full name: the baculoviral inhibitor of apoptosis repeat-containing 5) protein. While goniothalamin has demonstrated inhibitory action against transcription of the *BIRC5* gene at the RNA level, thus subjecting NCI-H460 cells to DNA damage (Semprebon et al., 2014).

In our previous work, styryllactone compounds were extracted from *Goniothalamus griffithii* (Annonaceae) and *Goniothalamus leiocarpus* (Annonaceae) (Mu et al., 1996; Li et al., 1997; Li et al., 1998; Mu et al., 1998; Mu et al., 1999a; Mu et al., 1999b; Mu et al., 2002; Mu et al., 2003; Mu et al., 2004). Their relative configurations were initially established on the basis of spectroscopic data (Li et al., 1997; Li et al., 1998; Mu et al., 1999a; Mu et al., 1999b; Mu et al., 2002; Mu et al., 2004).

In this study, the absolute configurations of several styryllactone compounds, first isolated in our laboratory, were determined by single-crystal X-ray diffraction analysis using Cu Kα radiation. In addition, we evaluated the effect of complexation of sytryllactones with HP-*β*-CD on their antitumor activity. The styryllactones displayed enhanced antitumor activity when complexd with HP-*β*-CD.

## Materials and Methods

### Materials

Dulbecco's modified Eagle's medium (DMEM), Roswell Park Memorial Institute 1640 (RPMI-1640) medium, minimum Eagle's medium (MEM), fetal bovine serum (FBS), and 0.25% trypsin–ethylenediaminetetraacetic acid (EDTA) were obtained from Life Technologies INC. (Grand Island, NY, USA). Trypan blue, penicillin, streptomycin, dimethyl sulfoxide (DMSO), VP-16 (Etoposide) and taxol were supplied by Sigma Chemical Co. (St. Louis, MO, USA). 3-(4,5-dimethylthiazol-2-yl)-2,5-diphenyl-tetrazolium bromide (MTT) was purchased from Molecular Probes Inc. (Eugene, OR, USA). Hydroxypopyl-*β*-cyclodextrin (HP-*β*-CD) was obtained from Nihon Shoukuhin Kako Co. Ltd. (Shibuya-ku, Tokyo, Japan). Phosphate buffer saline (PBS), annexin V fluorescein isothiocyanate (FITC)/propidium iodide (PI) Apoptosis Detection Kit, and caspase activity kit were purchased from Keygentec (Nanjing, Jiangsu, China). The water used in the experiments was obtained from the Milli-Q Water Purification System (MilliporeCorp., Billerica, MA, USA).

### Single-Crystal X-Ray Analysis

Data for diffraction intensity was obtained using a Bruker APEX-IICCD X-ray diffractometer (Bruker AXSGmbH, Karlsruhe, Germany) and graphite-monochromated Cu Kα radiation (λ= 1.54178 Å). Cell refinement and data reduction were performed with Bruker SAINT (Bruker AXS, GmbH, Karlsruhe, Germany). The absorption correction was determined semi-empirically from equivalent compounds. The structures were determined *via* direct methods using SHELXS-97 (Institute of Inorganic Chemistry of Georg-August-Universität Göttingen, Gottingen, Germany). Non–hydrogen atoms were anisotropically refined with SHELXL-97 (Non–hydrogen atoms of leiocarpin B were anisotropically refined with SHELXL-2014). Hydrogen atoms were located by geometry and positioned on the related atoms during refinements, with a temperature factor.

### Cell Culture and Assay

The human colon cancer SW1116 cell line, the human hepatocellular carcinoma SMMC-7721 cell line, the human gastric cancer SGC-7901 cell line, and the human hepatocellular carcinoma HepG2 cell line were kindly provided by Xiao-li Xu from the Cancer Center, Fudan University. The human embryonic kidney 293T (HEK293T) cell line was kindly donated by Professor You-hua Xie from Fudan University. These human cancer cell lines were cultured in DMEM medium or RPMI-1640 medium, whereas the HEK293T cell line was cultured in MEM medium. All of the cell lines were supplemented with 10% FBS, penicillin (100 U/ml), and streptomycin (100 mg/ml) under a humidified atmosphere of 5% CO_2_ at 37°C using a CO_2_ incubator (SANYO, Osaka, Japan). Cell count was assessed using the trypan blue dye exclusion method.

The antiproliferative effects of the treatments were evaluated using the MTT assay. Cells were seeded at a density of 5×10^3^ cells/well in 96-well plates (Corning, NY, USA). After attachment, the culture media were replaced with various concentrations of chemicals for 24 h. Then, the media in 96-well plates were incubated with MTT reagent (5 mg/ml) for 4 h at 37°C. Subsequently, the culture medium was discarded and 100 μl of DMSO was added to each well, to solubilize the formazan crystal formed. The absorbance of formazan crystal solution was determined at 570 nm on a Multiskan FC from Thermo Fisher Scientific Inc. (Waltham, MA, USA).

### Annexin V-FITC/PI Double Staining by Flow Cytometry

The growing cells were incubated in 24-well microplates (Corning, NY, USA) for 24 h. The cells were then treated with various concentrations of leiocarpin E-7′-monoacetate or taxol in humidified air with 5% CO_2_ at 37°C. After 36 h of incubation, the culture medium was discarded, and the cells were collected. For the apoptosis analysis, cells were suspended with 1×binding buffer (1×10^6^ cells/ml) and then labeled with annexin V-FITC/PI, as per the manufacturer's instructions (Keygentec, Nanjing, Jiangsu, China). The analysis of the samples was performed by flow cytometry (Becton-Dickinson Bioscience, San Jose, CA, USA), and the acquired data was analyzed by the CellQuest software (Becton-Dickinson Bioscience, San Jose, CA, USA).

### Caspase Activity

To evaluate the activity of caspases, cell lysates were prepared after their respective designated treatments. The incubation of the growing cells was carried out in 24-well microplates (Corning, NY, USA) for 24 h. The cells were then treated with different concentrations of leiocarpin E-7′-monoacetate under humidified air with 5% CO_2_ at 37°C. After 8 h of incubation, the culture medium was discarded, the cells collected and washed twice with PBS. The mixture was then centrifuged at 2,000 rpm for 5 min. The PBS supernatant was discarded and the cells (concentration, 5×10^6^ cells) were collected. To these cells, ice-cold lysis buffer (150~200 μl) was added. The mixture was placed on ice for 30 min, and then centrifuged (10,000 rpm, 1 min) at 4°C. The supernatant, containing lysed protein, was carefully aspirated and transferred to a new tube. The protein concentration was then measured in 2 μl of the supernatant using the Bradford method. The caspase assays were then performed in 96-well microtiter plates (Keygentec, Nanjing, Jiangsu, China) by incubating 10 μl of protein cell lysate per sample in 80 μl of reaction buffer (1% NP-40, 20 mM Tris-HCl (pH 7.5), 137 mM NaCl, and 10% glycerol) containing 10 μl of caspase substrate (2 mM). Lysates were incubated at 37°C for 4 h. Measuremet was done at 405 nm on a Multiskan FC from Thermo Fisher Scientific Inc. (Waltham, MA, USA). The detailed analysis procedure is described in the manufacturer's protocol (Keygentec, Nanjing, Jiangsu, China).

### Preparation of HP-*β*-CD Complex

A fixed quantity of the compound was weighed and evenly dispersed in an aqueous solution of HP-*β*-CD (molecular ratio of 1:2). The dispersion was equilibrated for 24 h at room temperature, under constant stirring. The supernatant was then lyophilized using a Christ Alpha1-2 Ld10 Freeze Dryer (Martin Christ Gefriertrocknungsanlagen GmbH, Osterode, Germany) to obtain the inclusion complex in a dry powder form. The content of styryllactones in the complex was determined using an ultraviolet (UV) spectrophotometer (Shimadzu, Kyoto, Japan). When the complexes were used in the antitumor test, we first prepared a solution of the compound, and then performed full wavelength scanning. Subsequently, we selected the maximum absorption wavelength of the compound as the detection wavelength. A 20-mg quantity of the complex was accurately weighed and placed in a 10-ml volumetric flask. It was then dissolved in 0.1 mol/L hydrochloric acid-acetonitrile-water (1:1:2), and the volume was recorded. The absorbance was measured at the detection wavelength. We calculated the total amount of compound (W1) in the complex, according to the standard equation obtained with a compound solution prepared with 0.1 mol/L hydrochloric acid-acetonitrile-water (1:1:2). Another 20 mg of the same complex was accurately weighed, and dissolved in 0.1 mol/L hydrochloric acid-acetonitrile (1:1) and placed in an ultrasound machine for 10 min. Subsequently, it was filtered and the filtrate was used to measure the absorbance at the detection wavelength. We calculated the free compound content (W2), according to the standard equation obtained with a compound solution prepared with 0.1 mol/L hydrochloric acid-acetonitrile (1:1). The difference between W1 and W2 represented the quantity of the compound that formed HP-*β*-CD complex in a 20-mg inclusion compound sample.

### Differential Scanning Calorimetry

The thermal characteristics of the raw material, HP-*β*-CD, the physical mixtures, and the complexes were determined using a differential scanning calorimeter (DSC; NETZSCH DSC system), equipped with a computerized data station (TA-50WS/PC, Selb, Bavaria, Germany). Samples were accurately weighed in a crimped aluminum pan and heated under an inert atmosphere of nitrogen. An empty pan sealed in the same manner, was used as a reference. The scanning rate was 10°C/min, and the scanning temperature ranged between 30°C and 400°C.

### Statistical Analysis

All data were expressed as means ± standard error of the mean (SEM) and were analyzed using two-tailed Student's *t*-tests. Statistical analyses were performed using SPSS 16.0 (SPSS Inc., Chicago, IL, USA). A value of *p* < 0.05 was considered statistically significant.

## Results

### Determination of the Absolute Configurations of Styryllactones

**Figure 2:**
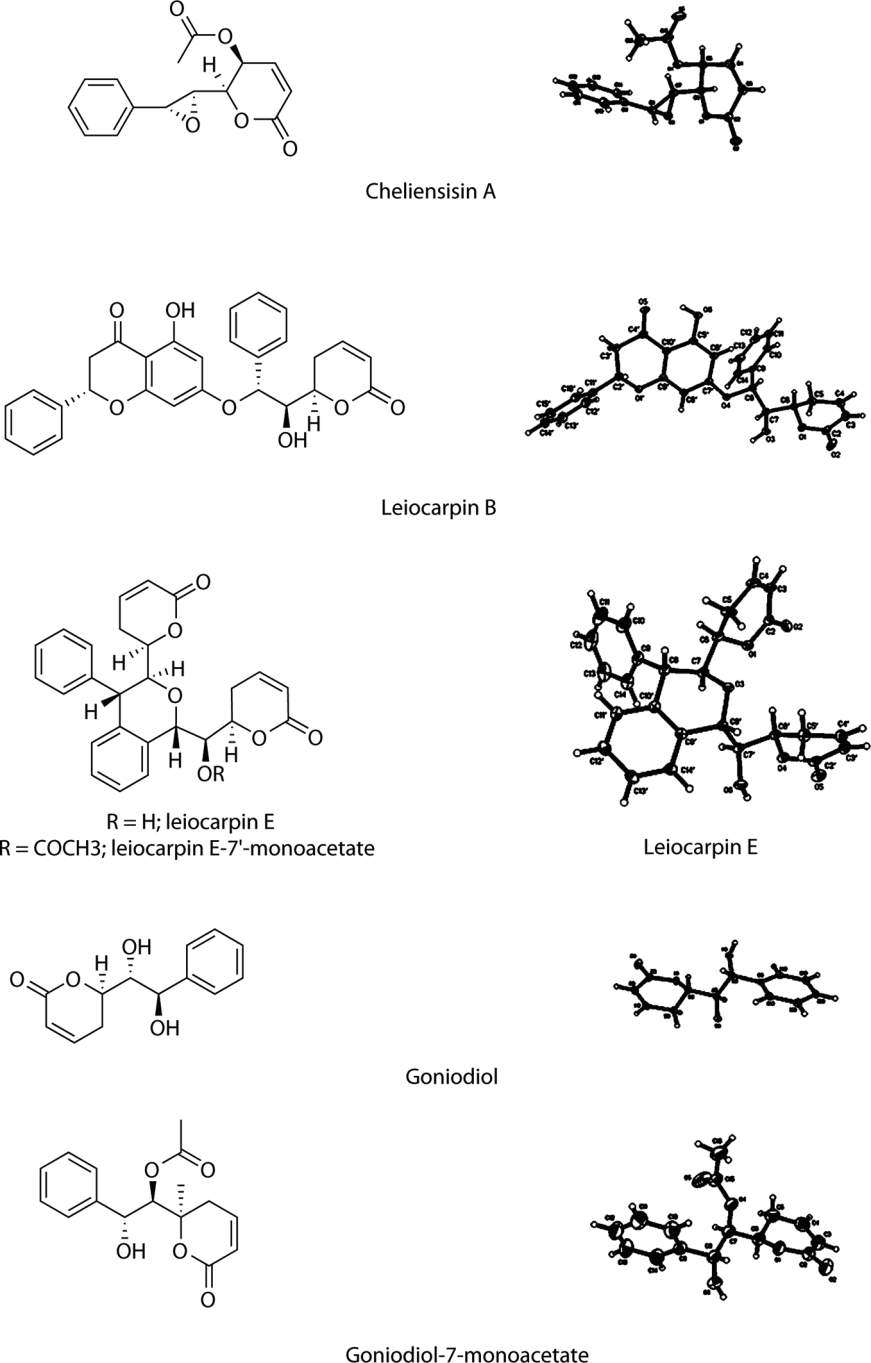
Structure and X-ray crystallography of the styryllactones.

Single-crystal X-ray diffraction analysis, using Cu Kα radiation, was used to identify the absolute configurations of styryllactone compounds (**Figure 2**). Based on single-crystal X-ray diffraction, the structure of cheliensisin A was established as (2*R*,3*S*)-6-oxo-2-((2*S*,3*R*)-3-phenyloxiran-2-yl)-3,6-dihydro-2*H*-pyran-3-yl acetate, and the absolute configuration of cheliensisin A was defined as 5*S*, 6*S*, 7*S*, 8*R*. The structure of leiocarpin B was established as (*S*)-5-hydroxy-7-((1*R*,2*R*)-2-hydroxy-2-((*R*)-6-oxo-3,6-dihydro-2*H*-pyran-2-yl)-1-phenylethoxy)-2-phenylchroman-4-one, and the absolute configuration of leiocarpin B was defined as 2′*S*, 6*R*, 7*S*, 8*R*. The structure of leiocarpin E was established as (*R*)-6-((*R*)-hydroxy((1*S*,3*S*,4*S*)-3-((*R*)-6-oxo-3,6-dihydro-2*H*-pyran-2-yl)-4-phenylisochroman-1-yl)methyl)-5,6-dihydro-2*H*-pyran-2-one, and the absolute configuration of leiocarpa E was defined as 6*R*, 6′*R*, 7*S*, 7′S, 8*S*, 8′*S*. Both goniodiol and goniodiol-7-monoacetate were identified as 6*R*, 7*R*, 8*R*. The structure of goniodiol was established as (*R*)-6-((1*R*,2*R*)-1,2-dihydroxy-2-phenylethyl)-5,6-dihydro-2*H*-pyran-2-one. The absolute configuration of leiocarpin E-7′-monoacetate, which is the acetate of leiocarpin E, was consistent with that of leiocarpin E.

The crystal data of cheliensisin A (C_15_H_14_O_5_) were as follows (detailed parameter shown in **Supplementary Table 1**): molecular weight (MW) = 274.26; orthorhombic, space group *P*2_1_2_1_2_1_; a = 7.0297 (10) Å, b = 11.0918 (10) Å, c = 17.5287 (3) Å; *α*= 90°, *β*= 90°, *γ* = 90°; V = 1366.75 (3) Å^3^, T = 123 (2) K; Z = 4; *D*_calc_ = 1.333 g·cm^−3^; index range: −8 ≤ h ≤ 8, −13 ≤ k ≤ 13, −18 ≤ l ≤ 20; absorption coefficient = 0.842 mm^−1^; completeness: 99.9%; *F*(0 0 0) = 576; GOF (goodness of fit) = 1.068. A colorless prismatic crystal with approximate dimensions of 0.21 mm × 0.15 mm × 0.14 mm was chosen and mounted on a Bruker APEX-II CCD diffractometer. The *θ* range for data collection was 4.72°–67.48°. A total of 8,080 reflections were collected, of which 2,435 were unique (*R*(int) = 0.0300) and 2,418 were considered observed (*I*> 2σ(*I*)). The maximum and minimum transmissions were 0.7456 and 0.3475. The refinement method was full-matrix least squares on F2. Data/restraints/parameters were 2435/0/183. The final *R* values were *R*_1_ = 0.0272 and *wR*_2_ = 0.0670 for 2,418 observed reflections, and *R*_1_ = 0.0274 and *wR*_2_ = 0.0673 for all observations. A full list of crystallographic data was deposited at the Cambridge Crystallographic Data Center, CCDC 1007949.

The crystal data of leiocarpin B (C_28_H_24_O_7_) were as follows (detailed parameter shown in **Supplementary Table 2**): MW= 472.47; monoclinic, space group *C*2; a = 21.0501 (4) Å, b = 7.9013 (10) Å, c = 16.4209 (3) Å; *α* = 90°, *β* = 121.2160 (10)°, *γ* = 90°; V = 2335.75 (7) Å^3^, T = 140 (2) K; Z = 4; *D*_calc_ = 1.344 g·cm^−3^; index range: −25 ≤ h ≤ 25, −9 ≤ k ≤ 9, −19 ≤ l ≤ 19; absorption coefficient = 0.798 mm^−1^; completeness: 98.2%; *F*(0 0 0) = 992; GOF (goodness of fit) = 1.079. A colorless block crystal, with approximate dimensions of 0.35 mm × 0.26 mm × 0.20 mm, was chosen and mounted on a Bruker APEX-II CCD diffractometer. The *θ* range for data collection was 3.15°–69.15°. A total of 6,301 reflections were collected, of which 3,373 were unique (*R*(int) = 0.0432) and 3,343 were considered observed (*I*> 2σ(*I*)). The maximum and minimum transmissions were 0.7532 and 0.4386. The refinement method was full-matrix least squares on F2. Data/restraints/parameters were 3373/1/319. The final *R* values were *R*_1_ = 0.0419 and *wR*_2_ = 0.1082 for 3,343 observed reflections, and *R*_1_ = 0.0421 and *wR*_2_ = 0.1089 for all observations. A full list of crystallographic data was deposited at the Cambridge Crystallographic Data Center, CCDC 1008036.

The crystal data of leiocarpin E (C_26_H_24_O_6_) were as follows (detailed parameter shown in **Supplementary Table 3**): MW= 432.45; orthorhombic, space group *P*2_1_2_1_2_1_; a = 9.7380 (10) Å, b = 11.1611 (2) Å, c = 20.3823 (3) Å; *α* = 90°, *β* = 90°, *γ* = 90°; V = 2215.29 (6) Å^3^, T = 123 (2) K; Z = 4; *D*_calc_ = 1.297 g·cm^−3^; index range: −11 ≤ h ≤ 11, −13 ≤ k ≤ 13, −24 ≤ l ≤ 24; absorption coefficient = 0.754 mm^−1^; completeness: 99.5%; *F*(0 0 0) = 912; GOF (goodness of fit) = 1.058. A colorless prismatic crystal, with approximate dimensions of 0.19 mm × 0.15 mm × 0.12 mm, was chosen and mounted on a Bruker APEX-II CCD diffractometer. The *θ* range for data collection was 4.34°–65.50°. A total of 12,242 reflections were collected, of which 3,746 were unique (*R*(int) = 0.0432) and 3,700 were considered observed (*I*> 2σ(*I*)). The maximum and minimum transmissions were 0.9129 and 0.8706. The refinement method was full-matrix least squares on F2. Data/restraints/parameters were 3746/0/291. The final *R* values were *R*_1_ = 0.0426 and *wR*_2_ = 0.1191 for 3,700 observed reflections, and *R*_1_ = 0.0430 and *wR*_2_ = 0.1195 for all observations. A full list of crystallographic data was deposited at the Cambridge Crystallographic Data Center, CCDC 1007951.

The crystal data of goniodiol (C_13_H_14_O_4_) were as follows (detailed parameter shown in **Supplementary Table 4**): MW = 234.24; orthorhombic, space group *P*2_1_2_1_2_1_; a = 9.2443 (2) Å, b = 9.7650 (2) Å, c = 13.0267 (3) Å; *α* = 90°, *β* = 90°, *γ* = 90°; V = 1175.93 (4) Å^3^; T = 140 (2) K; Z = 4; *D*_calc_ = 1.323 g·cm^−3^; index ranges: −10 ≤ h ≤ 10, −10 ≤k ≤ 11, −14 ≤ l ≤ 15; absorption coefficient = 0.814 mm^−1^; completeness: 98.1%; *F*(0 0 0) = 496; GOF (goodness of fit) = 1.141. A colorless block crystal, with approximate dimensions of 0.35 mm × 0.26 mm × 0.22 mm, was chosen and mounted on a Bruker APEX-II CCD diffractometer. The *θ* range for data collection was 5.66°–69.43°. A total of 5,322 reflections were collected, of which 2,070 were unique (*R*(int) = 0.0369) and 2,058 were considered observed (*I*> 2σ(*I*)). The maximum and minimum transmissions were 0.7532 and 0.5891. The refinement method was full-matrix least squares on F2. Data/restraints/parameters were 2070/0/157. The final *R* values were *R*_1_ = 0.0404 and *wR*_2_ = 0.1007 for 2,058 observed reflections, and *R*_1_ = 0.0405 and *wR*_2_ = 0.1008 for all observations. A full list of crystallographic data was deposited at the Cambridge Crystallographic Data Center, CCDC 1008511.

The crystal data of goniodiol-7-monoacetate (C_15_H_16_O_5_) were as follows (detailed parameter shown in **Supplementary Table 5**): MW= 276.28; triclinic, space group *P*1; a = 5.4547 (5) Å, b = 8.8394 (7) Å, c = 15.3120 (13) Å; *α* = 94.379 (6)°, *β* = 91.949 (5)°, *γ* = 105.106 (6)°; V = 709.58 (11) Å^3^; T = 296 (2) K; Z = 2; *D*_calc_ = 1.293 g·cm^−3^; index ranges: −6 ≤ h ≤ 6, −10 ≤ k ≤ 10, −18 ≤ l ≤ 15; absorption coefficient = 0.811 mm^−1^; completeness: 94.0%; *F*(0 0 0) = 292; GOF (goodness of fit) = 1.042. A colorless block crystal, with approximate dimensions of 0.2 mm × 0.12 mm × 0.05 mm, was chosen and mounted on a Bruker APEX-II CCD diffractometer. The *θ* range for data collection was 2.90°–69.66°. In total, 5,692 reflections were collected, of which 3,307 were unique (*R*(int) = 0.0419) and 3,046 were considered observed (*I*> 2σ(*I*)). The maximum and minimum transmissions were 0.7532 and 0.4727. The refinement method was full-matrix least squares on F2. Data/restraints/parameters were 3307/3/365. The final *R* values were *R*_1_ = 0.0468 and *wR*_2_ = 0.1251 for 3,046 observed reflections, and *R*_1_ = 0.0505 and *wR*_2_ = 0.1309 for all observations. A full list of crystallographic data was deposited at the Cambridge Crystallographic Data Center, CCDC 1442700.

### Styryllactones Inhibit the Proliferation of Tumor Cell Lines

**Figure 3:**
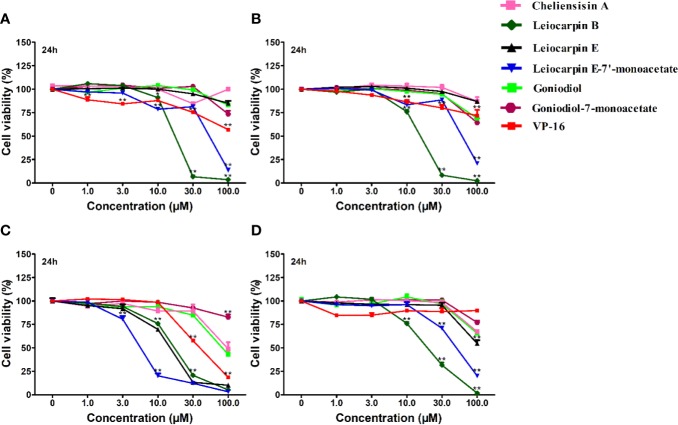
Styryllactones inhibited proliferation of tumor cell lines. The cells (5×10^4^ cells/ml) were cultured in the absence or presence of various compounds (1.0, 3.0, 10.0, 30.0, 100.0 μM) for 24 h. VP-16 (1.0, 3.0, 10.0, 30.0, 100.0 μM) were used as a positive control. **(A)** SGC-7901 cell line; **(B)** SMMC-7721 cell line; **(C)** SW1116 cell line; **(D)** HepG2 cell line. Data are means ± standard error of the mean (SEM) (*n*=3). Results are representative of three separate experiments. *P<0.05, **P<0.01 compared with the group (cell viability in 0 μM).

The *in vitro* cytotoxic activity of styryllactones was evaluated in four human tumor cell lines by the MTT assay (**Figure 3**). VP-16 was chosen as the positive control, because it is one of the most widely used cancer chemotherapy agents to treat many kinds of cancers, and it could induce apoptosis of cancer cells by acting as a toposiomerase II inhibitor (Berger et al., 1996; Chiu et al., 2005). The results showed that cheliensisin A, goniodiol and goniodiol-7-monoacetate had no cytotoxic effect on the SGC-7901, SMMC-7721 and HepG2 cell lines since the IC_50_ of these compounds were greater than 100 μM. In contrast, leiocarpin B, leiocarpin E, and leiocarpin E-7′-monoacetate showed excellent cytotoxicity against the SW1116 cell line, when their concentrations were 30 μM, the cell viability of SW1116 cells was significantly less (*p* < 0.01) than the normal group (cell viability in 0 μM). The highest cytotoxic effect against SW1116 cells was demonstrated by leiocarpin E-7′-monoacetate, while cheliensisin A and goniodiol-7-monoacetate showed relatively lower inhibitory effect (**Table 1**). Thus, it was concluded that the human colon cancer SW1116 cell line was much more sensitive to the cytotoxic effect of styryllactones.

**Table 1:** Styryllactones inhibited proliferation of cell lines.

	IC_50_ (μM)				
	SGC-7901	SMMC-7721	SW1116	HepG2	HEK293T
**Cheliensisin A**	>100	>100	93.18 ± 0.78	>100	>100
**Leiocarpin B**	18.21 ± 0.88	15.45 ± 1.11	17.50 ± 0.69	19.34 ± 1.42	>100
**Leiocarpin E**	>100	>100	15.11 ± 1.87	>100	>100
**Leiocarpin E-7′-monoacetate**	45.03 ± 4.27	61.71 ± 10.14	6.73 ± 0.89	50.11 ± 3.25	>100
**Goniodiol**	>100	>100	80.05 ± 4.16	>100	>100
**Goniodiol-7-monoacetate**	>100	>100	>100	>100	>100
***VP-16***	*>100*	*>100*	*41.87 ± 0.98*	*>100*	*>100*

Data are means ± SEM (n=3). Results are representative of three separate experiments.

### Cytotoxic Effect of Styryllactones Against the Human Normal Cell Line HEK293T

**Figure 4:**
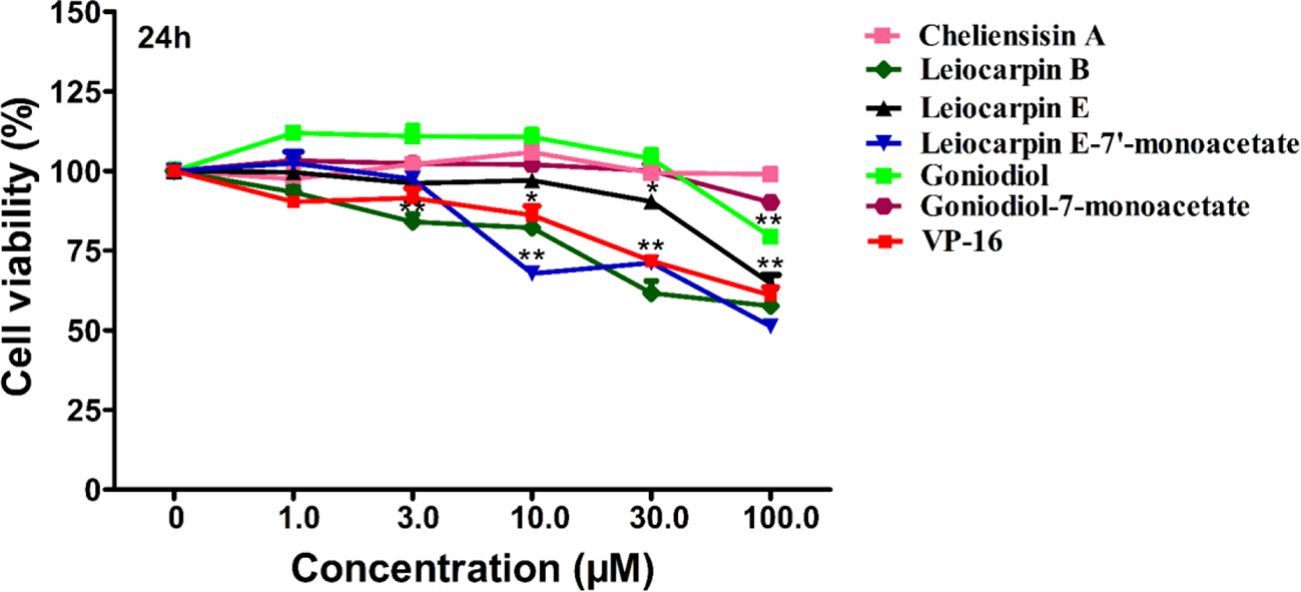
Cytotoxic effect of styryllactones against the human normal cell line human embryonic kidney 293T (HEK293T). The cells (5×10^4^ cells/ml) were cultured in the absence or presence of styryllactone compounds (1.0, 3.0, 10.0, 30.0, 100.0 μM) for 24 h. VP-16 (1.0, 3.0, 10.0, 30.0, 100.0 μM) were used as a positive control. Data are means ± standard error of the mean (SEM) (*n*=3). Results are representative of three separate experiments. *P < 0.05, **P < 0.01 compared with the group (cell viability in 0 μM).

The human embryonic kidney 293T (HEK293T) cell line is used as a normal human cell line in various biological experiments (Chen et al., 2016; Vemuri et al., 2019). In this study, we investigated the *in vitro* cytotoxic effect of styryllactones (concentration ranging from 0 and 100 μM) against HEK293T cells (**Figure 4**). The results showed that the IC_50_ values of all the compounds against HEK293T cells were above 100 μM (**Table 1**). This indicated that styryllactones did not show cytotoxicity against normal human cell line.

### Leiocarpin E-7ʹ-Monoacetate Induces the Early Apoptosis of SW1116 Cells

**Figure 5:**
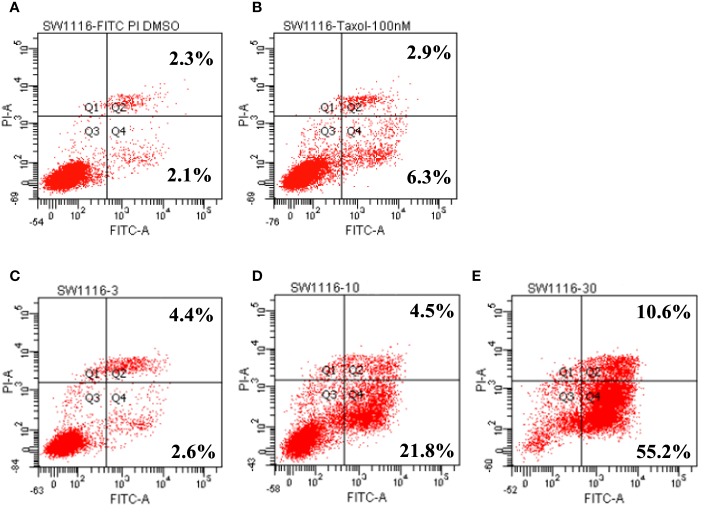
Leiocarpin E-7′-monoacetate induced early apoptosis. The annexin V fluorescein isothiocyanate (FITC)/propidium iodide (PI) staining assay was analyzed by flow cytometry. SW 1116 cells were cultured with chemicals for 36 h: **(A)** vehicle; **(B)** Taxol (100 nM); **(C)** leiocarpin E-7′-monoacetate (3 μM); **(D)** leiocarpin E-7′-monoacetate (10 μM); **(E)** leiocarpin E-7′-monoacetate (30 μM).

In the *in vitro* cytotoxicity experiments, SW1116 cells were found to be sensitive to leiocarpin E-7′-monoacetate. To further evaluate the apoptotic activity of leiocarpin E-7′-monoacetate, the cells were treated with various concentrations of leiocarpin E-7′-monoacetate and then analyzed by flow cytometry. Taxol was chosen as the positive control, because it could stabilize microtubules, and subsequently cause cell apoptosis by arresting the cell cycle at G2/M (Ruden and Puri, 2013; Luo et al., 2015). The results showed that the early apoptosis rates were 2.6%, 21.8%, and 55.2% when the concentration of leiocarpin E-7′-monoacetate was 3, 10, and 30 μM, respectively (**Figure 5**). In summary, the results indicated that leiocarpin E-7'-monoacetate was able to induce the apoptosis of SW1116 cells in a concentration-dependent manner.

### Leiocarpin E-7ʹ-Monoacetate-Induced Apoptosis is Caspase-Dependent

**Figure 6:**
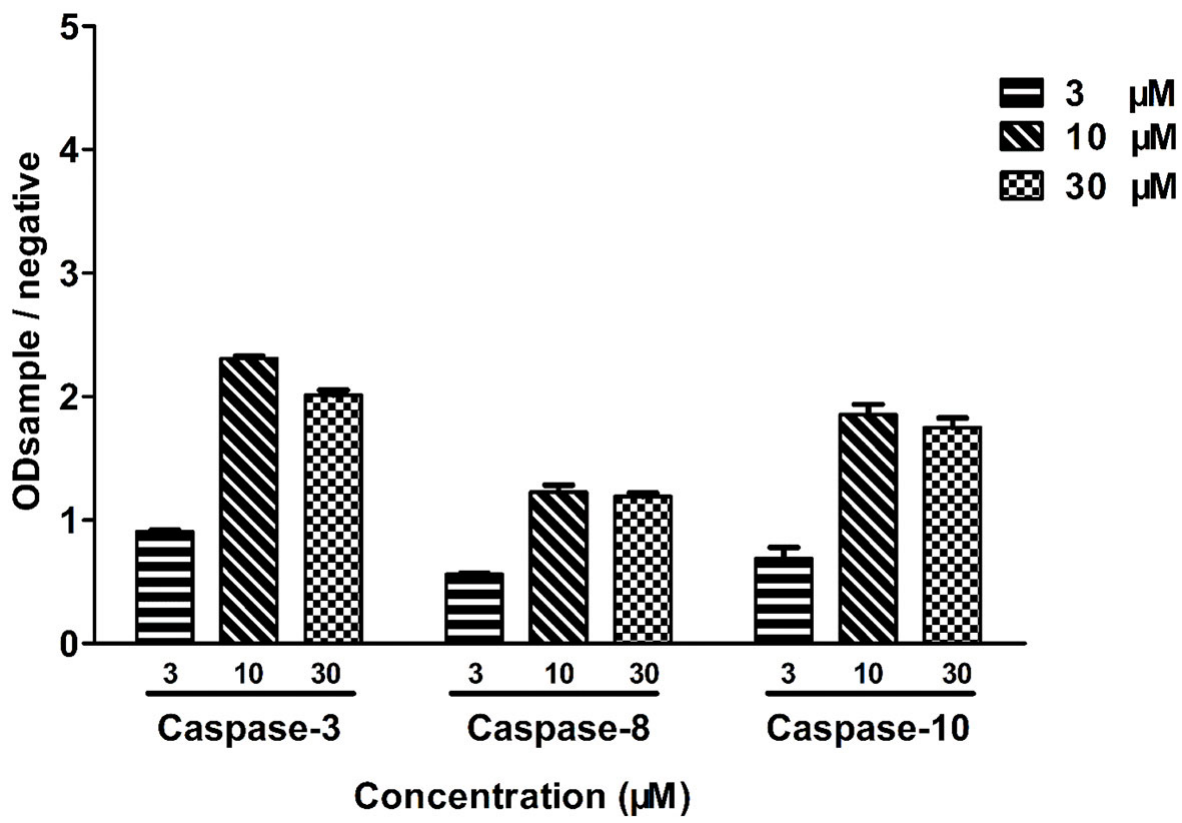
Leiocarpin E-7′-monoacetate induced-apoptosis was caspase-dependent. SW1116 cells were treated with leiocarpin E-7′-monoacetate (3, 10, and 30 μM) for 8 h. Data are presented as means ± SEM (*n*=3).

To further examine the cytotoxicity induced by leiocarpin E-7′-monoacetate, the activity of the apoptosis-associated protease was studied, using an enzyme activity assay kit. The results showed that different kinds of caspase proteases were activated as the concentration of leiocarpin E-7′-monoacetate was increased (**Figure 6**). When the concentration was 3 μM, the ration of OD_sample_ to OD_negative_ was not greater than 1, as it indicated that the caspase enzymes were not activated. However, when the concentration was increased to 10 and 30 μM, the ration was greater than 1, as it demonstrated that the activation of caspase enzymes increased significantly.

### Identification of Complexes by Differential Scanning Calorimetry

**Figure 7:**
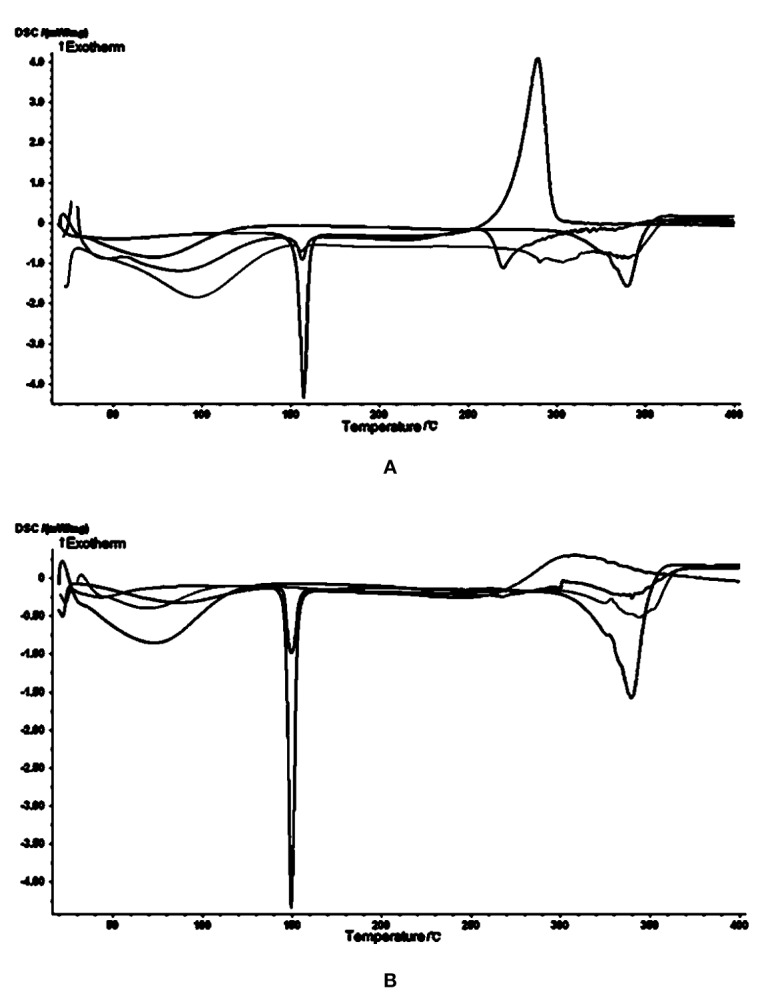
Images from the differential scanning calorimetry (DSC) analysis. Green line: hydroxypropyl-*β*-cyclodextrin (HP-*β*-CD); blue line: styryllactone only; brown line: physical mixture; pink line: complexes of HP-*β*-CD with **(A)** cheliensisin A and **(B)** gonidiol-7-monoacetate.

In the cytotoxicity experiments, cheliensisin A and gonidiol-7-monoacetate showed no effects on tumor growth. This could be attributed to the poor water solubility of these compounds. To improve the solubility, the styryllactones HP-*β*-CD complexes were synthesized *via* the saturated aqueous solution method. A DSC analysis was then carried out for HP-*β*-CD, styryllactone, a styryllactone/HP-*β*-CD physical mixture, and the styryllactone/HP-*β*-CD complex (**Figure 7**).

The raw cheliensisin A (**Figure 7A**) had a sharp endothermic peak and a sharp exothermic peak near 150°C and 300°C, respectively. HP-*β*-CD had a broad endotherm near 350°C, which was also present in the mixture. However, in the spectrum of the complex, the characteristic peak of cheliensisin A disappeared. This indicated that the compound penetrated into the cyclodextrin cavity and replaced the water molecules.

The raw goniodiol-7-monostearate monomer (**Figure 7B**) had a sharp endothermic peak at 150°C, which were also present in the mixture. The DSC curves of the complex showed that the characteristic peaks of the goniodiol-7-monostearate monomer disappeared, which confirmed the formation of the styryllactone complex with HP-*β*-CD.

### Increased Cytotoxicity Activity of the Complex

**Figure 8:**
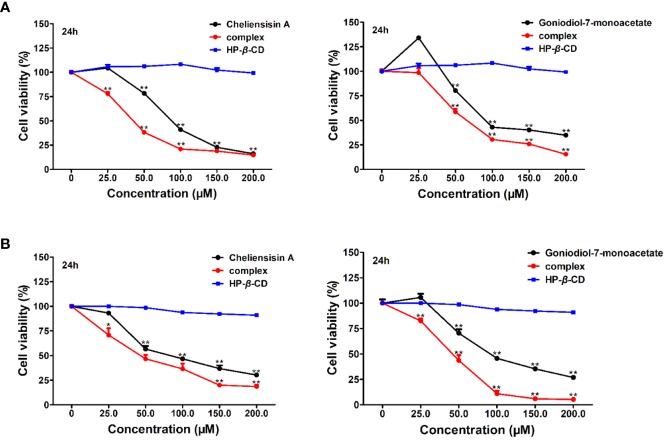
Cytotoxic activity was enhanced by hydroxypropyl-*β*-cyclodextrin (HP-*β*-CD) complexes of styryllactones. SMMC-7721 cells **(A)** and SW1116 cells **(B)** (5×10^4^ cells/ml) were cultured respectively in the absence or presence of various compounds and complexes (25.0, 50.0, 100.0, 150.0, 200.0 μM) for 24 h: cheliensisin A, cheliensisin A/HP-*β*-CD complex and HP-*β*-CD; gonidiol-7-monoacetate, goniodiol-7-monoacetate/HP-*β*-CD complex and HP-*β*-CD. Data are means ± SEM (*n*=3). Results are representative of three separate experiments. *P<0.05, **P<0.01 compared with the group (cell viability in 0 μM).

To examine whether complexation of styryllactone with HP-*β*-CD resulted in an enhancement of antitumor activity against different human cell lines, cytotoxicity experiments were evaluated in SMMC-7721 and SW1116 cell lines, using the MTT assay (**Figure 8**).

The results showed that the styryllactone/HP-*β*-CD complexes were significantly more cytotoxic than their respective pristine form (*P* < 0.01), while HP-*β*-CD did not show any inhibition of the growth of the two cell lines (**Table 2**). The IC_50_ of cheliensisin A and goniodiol-7-monoacetate were reduced by 45% and 55%, respectively against the SW1116 cell line. Similarly, the IC_50_ values of the two compounds were reduced by 58% and 34%, respectively against the SMMC-7721 cell line.

**Table 2:** Cytotoxic activity of styryllactones increased after complexation with Hydroxypopyl-β-cyclodextrin (HP-*β*-CD).

	IC_50_ (μM)					
	SW1116		SMMC-7721		HEK293T	
	Compound	Complex	Compound	Complex	Compound	Complex
**Cheliensisin A**	93.18 ± 0.78	51.44 ± 4.78^**^	110.90 ± 2.41	46.91 ± 1.60^**^	>200	98.46 ± 3.00^**^
**Goniodiol-7-monoacetate**	102.14 ± 4.15	46.48 ± 3.25^**^	115.54 ± 3.97	75.76 ± 4.19^**^	>200	35.02 ± 0.63^**^

Data are means ± SEM (n=3). Results are representative of three separate experiments. **P < 0.01 compared with the compound group.

### Cytotoxicity Activity Against HEK293T Cells is Increased by the Complex

Based on the results, it was observed that the complexes demonstrated enhanced cytotoxic effect against tumer cell lines, when compared with the styryllactone compounds. To study whether there is obviously enhanced cytotoxicity of complex in normal cell lines, the human normal cell line HEK293T was treated with styryllactone HP-*β*-CD complexes.

**Figure 9:**
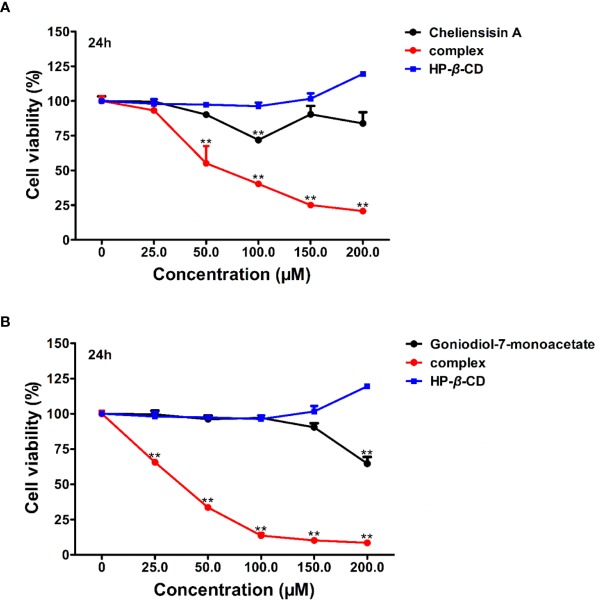
Cytotoxic activity of hydroxypropyl-*β*-cyclodextrin (HP-*β*-CD) complexes of styryllactones was increased against human embryonic kidney 293T (HEK293T) cells. The cells (5×10^4^ cells/ml) were cultured respectively in the absence or presence of various compounds and complexes (25.0, 50.0, 100.0, 150.0, 200.0 μM) for 24 h: **(A)** cheliensisin A, cheliensisin A/HP-*β*-CD complex and HP-*β*-CD; **(B)** gonidiol-7-monoacetate, goniodiol-7-monoacetate/HP-*β*-CD complex and HP-*β*-CD. Data are means ± SEM (*n*=3). Results are representative of three separate experiments. **P<0.01 compared with the group (cell viability in 0 μM).

**Figure 9** suggested that the complexes showed significantly higher cytotoxic activity than the compounds against HEK293T cell line (*P* < 0.01). For example, the IC_50_ of cheliensisin A complex and goniodiol-7-monoacetate complex were 98.46 ± 3.00 and 35.02 ± 0.63 μM, respectively (**Table 2**). When the cells were treated with the pristine forms of cheliensisin A and goniodiol-7-monoacetate, the IC_50_ values obtained were beyond 200 μM. In addition, HP-*β*-CD did not show any inhibitory activity against the HEK293T cell line (**Figure 9**).

## Discussion

Styryllactones are a series of secondary metabolites isolated from the genus *Goniothalamus*, which generally contains 1 benzene ring and 1 unsaturated lactone ring (Bermejo et al., 1997; Bermejo et al., 1998; Cao et al., 1998; Bermejo et al., 1999; Hu et al., 1999; Mu et al., 1999a; Peris et al., 2000; Lan et al., 2005; Liou et al., 2014). In our previous work, styryllactone compounds were isolated from *Goniothalamus griffithii* (Annonaceae) and *Goniothalamus leiocarpus* (Annonaceae) (Mu et al., 1996; Li et al., 1997; Li et al., 1998; Mu et al., 1998; Mu et al., 1999a; Mu et al., 1999b; Mu et al., 2002; Mu et al., 2003; Mu et al., 2004), and the absolute configuration of leiocarpin B was determined by Mosher ester method (Mu et al., 2002). In this paper, the absolute configuration of five styryllactone compounds were determined by single-crystal X-ray diffraction analysis, using Cu Kα radiation.

Studies have shown that a number of styryllactones demonstrated cytotoxicity against various human cancer cell lines. For example, 7-acetylaltholactone had cytotoxicity against KB (oral epidermoid carcinoma cell line), HepG2 (liver cancer), and MCF7 (breast carcinoma) cell lines with IC_50_ values of 13.1, 23.7, and 60.2 μM, respectively (Trieu et al., 2014). Goniothalamin could inhibit the growth of RT4 cell line (urinary bladder) (Yen et al., 2014), HL-60 cell line (leukemia) (Ali et al., 1997), HGC-27 cell line (colon gastric) (Ali et al., 1997), HT-29 cell line (colon gastric) (Vendramini-Costa et al., 2016). Among the researches, cytotoxicity and apoptosis were induced (Ali et al., 1997; Yen et al., 2014; Vendramini-Costa et al., 2016), and caspase-3, -8 and -9 activation also occurred, suggesting caspase-dependent apoptotic pathway by other styryllactone compounds (Vendramini-Costa et al., 2016). However, the cytotoxicity and mechanism of the styryllactone compounds, first isolated in our laboratory, have not been studied in SMMC-7721, HepG2, SW1116 and SGC-7901 cell lines. Besides, SMMC-7721 cells (Kim, 2009), HepG2 cells (Li et al., 2019), SW1116 cells (Gu et al., 2018) and SGC-7901 (Wang et al., 2018) were all reported to be studied in apoptosis-induced research *via* activaing caspase proteas, indicating that they were able to be used in our mechanism research. As a result, to evaluate whether the styryllactones isolated could resulte in cytotoxicity and apoptosis *via* caspase-3, -8 and -9 activation, these four human cell lines were choosen in our research.

It was found that leiocarpin B, leiocarpin E, and leiocarpin E-7'-monoacetate had significant cytotoxic activities against human colon cancer SW1116 cell line. Further, the results of Annexin V-FITC/PI double staining showed that leiocarpin E-7'-monoacetate induced apoptosis of SW1116 cells in a concentration-dependent manner (**Figure 5**).

In mammalian cells, there are two major apoptotic pathways: the first one involves a signal from the mitochondria, while the second relies on signal transduction through death receptors. Studies have shown that caspase-8 is activated through a death receptor-mediated pathway and cleavage of caspase-9 plays a key role in mitochondria apoptotic pathway. Caspase-3 is the key executive molecule of the apoptotic signal (Dodson et al., 2013; Vendramini-Costa et al., 2016). We observed the occurrence of apoptosis at the cellular level. Subsequently, we investigated whether the activity of caspase enzymes was affected owing to the intervention of leocarpin E-7'-monoacetate at the enzyme level. It was observed that caspase-3, -8 and -9 showed activation at different concentrations of leocarpin E-7'-monoacetate (**Figure 6**). When the concentration of leocarpin E-7'-monoacetate was 3 μM, in the caspase activity test the ration of OD_sample_ to OD_negative_ was not greater than 1, while the apoptosis rate is low. When the concentration was increase to 10 and 30 μM, the apoptosis rate also increased.

However, cheliensisin A, goniodiol, and goniodiol-7-monoacetate showed poor *in vitro* activity. The application of styryllactones as antitumor agent has been strongly impeded owing to their poor water solubility (Zhao et al., 2008; Deng et al., 2011). In a study by Zhao et al, the solubility of cheliensisin A was improved by formulating it as a lyophilized submicron emulsion intravenous injection (Zhao et al., 2008). However, the process is complicated and expensive. In this study, we prepared inclusion complexes of styryllactones using HP-*β*-CD and evaluated the cytotoxic activity of the complex.

HP-*β*-CD is commonly used to enhance the water solubility of poorly soluble compounds. It contains a hydrophilic exterior surface and a nonpolar interior cavity. This structure allows HP-*β*-CD to act as a carrier that can encapsulate a poorly water-soluble compound in the internal area, thereby increasing the solubility of the compound (Kaur et al., 2014; Wang et al., 2014a). In the pharmaceutical industry, HP-*β*-CD is used to increase drug stability, improve bioavailability, and reduce side effects owing to its low surface activity, low hemolytic activity, and lack of muscle irritation. Thus, it is widely used as an injection solubilizer and pharmaceutical excipient (Kryjewski et al., 2015).

Differential Scanning Calorimetry (DSC) is a suitable thermal analysis technique used in the pharmaceutical industry for determining the purity, polymorphic forms, and melting point of a sample (Demetzos, 2008). In addition, DSC can provide detailed information about both the physical and energetic properties of a substance (Green et al., 2020). The results showed that apart from the characteristic peaks of styryllactones and HP-*β*-CD, no other endothermic or exothermic peak was observed (**Figure 7**). In addition, cyclodextrin is a cyclic oligosaccharide composed of covalently-linked glucopyranose rings, which can assist in increasing the solubility of hydrophobic drugs by forming water-soluble inclusions (Brewster and Loftsson, 2007). HP-*β*-CD is a chemically modified derivate of cyclodextrin that has a higher solubility in water and can be safely used as a complexing and solubilizing excipient in various drug administration routes (Peeters et al., 2002). For example, Al- Qubaisi et al. developed an inclusion complex of thymoquinone and HP-*β*-CD in order to improve solubility and bioactivity of thymoquinone. In their work, they proved that the entire thymoquinone molecule was entrapped in the HP-*β*-CD cavity and that the molecule was not degraded by the complexation (Al-Qubaisi et al., 2019). As a result, we demonstrated that the cyclodextrin complexation did not degrade the molecular structure.

It was observed that the antitumor effect of styryllactones complexed with HP-*β*-CD was significantly enhanced. From the experiments, the IC_50_ of the cheliensisin A complex was 51.44 ± 4.78 μM and 46.91 ± 1.60 μM against SW1116 cells and SMMC-7721 cells, respectively. The IC_50_ of goniodiol-7-monoacetate complex was 46.48 ± 3.25 μM and 75.76 **±** 4.19 μM, whereas the IC_50_ of the cheliensisin A monomer was 93.18 ± 0.78 μM and 110.90 ± 2.41 μM against SW1116 cells and SMMC-7721 cells, respectively. The IC_50_ of the goniodiol-7-monoacetate monomer was 102.14 ± 4.15 μM and 115.54 ± 3.97 μM, respectively (**Table 2**). HP-*β*-CD had no inhibitory effect on the growth of the two cell lines (**Figure 8**). The cytotoxicity of styryllactones and their complex was also evaluated in the human normal cell line HEK293T. It was observed that the HP-*β*-CD complexes showed greater cytotoxic activity than the styryllactone compounds against HEK293T cell line. This suggested that the complexes could also inhibit the growth of normal cell line. The IC_50_ of cheliensisin A complex and goniodiol-7-monoacetate complex were 98.46 ± 3.00 and 35.02 ± 0.63 μM, respectively (**Table 2**). Thus, there was a dose safety window when cheliensisin A complexes were treated against SW1116 cell line or SMMC-7721 cell line. In addition, there was a dose safety window when goniodiol-7-monoacetate complexes were treated against SW1116 cell line. However, the dose safety window was narrower when goniodiol-7-monoacetate complexes were treated against SMMC-7721 cell line. In this study, we focused on investigation of the method to enhance the cytotoxicity activity of styryllactones. HP-*β*-CD is known to increase the solubility of poorly water-soluble compounds (Kaur et al., 2014; Wang et al., 2014a). Thus, it was assumed that the enhanced antitumor effects of the complexes were partly because of the improved water solubility. The results from this study show that styryllactones have the potential to be developed as antitumor compounds. Next, we plan to obtain enough compounds, either by extraction from plants or chemical synthesis and then explore better compound structures based on these styryllactones. Their HP-*β*-CD complex will be developed and cytotoxicity will be studied. Subsequently, we intend to investigate the anticancer effect in the animal model using the styryllactones inclusion complex, and conduct efficient test methods including radiolabeling test and permeability test, to illustrate the exact mechanism underlying the improved potency of complexes toward tumor cell lines.

This paper described the identification of the absolute configurations of several styryllactones by single-crystal X-ray diffraction analysis using Cu Kα radiation. We synthesized styryllactones complexes with HP-*β*-CD for the first time. The *in vitro* antitumor experiments showed that the inhibitory activity of these complexes was greater than the respective pristine form of the compounds. These results suggest that the water solubility of styryllactones can be improved by complexation of styryllactones with HP-*β*-CD, which in turn, can enhance the antitumor activity of these compounds.

## Data Availability Statement

All datasets generated for this study are included in the article/**Supplementary Material**.

## Author Contributions

RM and QM contributed to the conception and design of the study. RM performed the experiments and wrote the first draft of the manuscript. J-TC assisted in the performance of experiments. X-LX donated the cells in the research. QM and X-YJ revised the manuscript. QM gave final approval of the version to be submitted. All authors read and approved the final manuscript.

## Conflict of Interest

The authors declare that the research was conducted in the absence of any commercial or financial relationships that could be construed as a potential conflict of interest.

## References

[B1] Ali A. M. Mackeen M. M. Hamid M. Aun Q. B. Zauyah Y. Azimahtol H. L. (1997). Cytotoxicity and electron microscopy of cell death induced by goniothalamin. Planta Med. 63, 81–83. 10.1055/s-2006-957611 9063100

[B2] Al-Qubaisi M. S. Rasedee A. Flaifel M. H. Eid E. E. M. Hussein-Al-Ali S. Alhassan F. H. (2019). Characterization of thymoquinone/hydroxypropyl-beta-cyclodextrin inclusion complex: Application to anti-allergy properties. Eur. J. Pharm. Sci. 133, 167–182. 10.1016/j.ejps.2019.03.015 30902654

[B3] Altobelli E. Lattanzi A. Paduano R. Varassi G. Di Orio F. (2014). Colorectal cancer prevention in Europe: burden of disease and status of screening programs. Prev. Med. 62, 132–141. 10.1016/j.ypmed.2014.02.010 24530610

[B4] Berger J. M. Gamblin S. J. Harrison S. C. Wang J. C. (1996). Structure and mechanism of DNA topoisomerase II. Nature 379, 225–232. 10.1038/379225a0 8538787

[B7] Bermejo A. Blazquez M. A. Serrano A. And Z. P. Cortes D. (1997). Preparation of 7-alkoxylated furanopyrones: Semisynthesis of (-)-etharvensin a new Styryllactone from Goniothalamus arvensis. J. Natural products 60, 1338–1340. 10.1021/np970346w

[B5] Bermejo A. Blazquez M. A. Rao K. S. Cortes D. (1998). Styryllactone from Goniothalamus arvensis. Phytochemistry 47, 1375–1380. 10.1016/S0031-9422(97)00770-X 9611830

[B6] Bermejo A. Blazquez M. A. Rao K. S. Cortes D. (1999). Styryllactone from the stem bark of Goniothalamus arvensis. Phytochemical Anal. 10, 127–131. 10.1002/(SICI)1099-1565(199905/06)10:3<127::AID-PCA451>3.0.CO;2-5

[B8] Brewster M. E. Loftsson T. (2007). Cyclodextrins as pharmaceutical solubilizers. Adv. Drug Delivery Rev. 59, 645–666. 10.1016/j.addr.2007.05.012 17601630

[B9] Cao S. G. Wu X. H. Sim K. Y. Tan B. K. H. Pereira J. T. Goh S. H. (1998). Styryllactone derivatives and alkaloids from Goniothalamus borneensis (Annonaceae). Tetrahedron 54, 2143–2148. 10.1016/S0040-4020(97)10422-7

[B10] Chen C. Wang C. C. Wang Z. Geng W. Y. Xu H. Song X. M. (2016). Cytotoxic activity of a synthetic deoxypodophyllotoxin derivative with an opened D-ring. J. Asian Nat. Prod Res. 18, 486–494. 10.1080/10286020.2015.1131679 27123550

[B11] Chiu C. C. Lin C. H. Fang K. (2005). Etoposide (VP-16) sensitizes p53-deficient human non-small cell lung cancer cells to caspase-7-mediated apoptosis. Apoptosis 10, 643–650. 10.1007/s10495-005-1898-8 15909125

[B12] Demetzos C. (2008). Differential Scanning Calorimetry (DSC): a tool to study the thermal behavior of lipid bilayers and liposomal stability. J. Liposome Res. 18, 159–173. 10.1080/08982100802310261 18770070

[B13] Deng X. Su J. Zhao Y. Peng L. Y. Li Y. Yao Z. J. (2011). Development of novel conformation-constrained cytotoxic derivatives of cheliensisin A by embedment of small heterocycles. Eur. J. Med. Chem. 46, 4238–4244. 10.1016/j.ejmech.2011.06.028 21775031

[B14] Dodson M. Darley-Usmar V. Zhang J. (2013). Cellular metabolic and autophagic pathways: traffic control by redox signaling. Free Radic. Biol. Med. 63, 207–221. 10.1016/j.freeradbiomed.2013.05.014 23702245 PMC3729625

[B15] Green S. P. Wheelhouse K. M. Payne A. D. Hallet J. P. Miller P.W. Bull J. A. (2020). Thermal Stability and Explosive Hazard Assessment of Diazo Compounds and Diazo Transfer Reagents. Org. Process Res. Dev 24, 67–84. 10.1021/acs.oprd.9b00422 31983869 PMC6972035

[B16] Gu Y. Y. Chen M. H. May B. H. Liao X. Z. Liu J. H. Tao L. T. (2018). Matrine induces apoptosis in multiple colorectal cancer cell lines in vitro and inhibits tumour growth with minimum side effects in vivo via Bcl-2 and caspase-3. Phytomedicine 51, 214–225. 10.1016/j.phymed.2018.10.004 30466620

[B17] Hu Z. B. Liao S. X. Mao S. L. Zhu H. P. Xu S. Liang H. Q. (1999). Studies on the chemical constituents of Goniothalamus griffithii Hook. f. et Thoms. Acta pharmacologica Sin. 34, 132–134. 10.16438/j .0513-4870.1999.02.012

[B18] Kaur M. Bhatia R. K. Pissurlenkar R. R. Coutinho E. C. Jain U. K. Katare O. P. (2014). Telmisartan complex augments solubility, dissolution and drug delivery in prostate cancer cells. Carbohydr Polym 101, 614–622. 10.1016/j.carbpol.2013.09.077 24299818

[B19] Kim S. H. (2009). The influence of finding meaning and worldview of accepting death on anger among bereaved older spouses. Aging Ment. Health 13, 38–45. 10.1080/13607860802154457 19197688

[B20] Kozovska Z. Gabrisova V. Kucerova L. (2014). Colon cancer: cancer stem cells markers, drug resistance and treatment. BioMed. Pharmacother. 68, 911–916. 10.1016/j.biopha.2014.10.019 25458789

[B21] Kryjewski M. Goslinski T. Mielcarek J. (2015). Functionality stored in the structures of cyclodextrin-porphyrinoid systems. Coordination Chem. Rev. 300, 101–120. 10.1016/j.ccr.2015.04.009

[B22] Lan Y. H. Chang F. R. Liaw C. C. Wu C. C. Chiang M. Y. Wu Y. C. (2005). Digoniodiol, deoxygoniopypyrone A, and goniofupyrone A: three new styryllactones from Goniothalamus amuyon. Planta Med. 71, 153–159. 10.1055/s-2005-837783 15729624

[B23] Lao V. V. Grady W. M. (2011). Epigenetics and colorectal cancer. Nat. Rev. Gastroenterol. Hepatol 8, 686–700. 10.1038/nrgastro.2011.173 22009203 PMC3391545

[B24] Levrier C. Sadowski M. C. Nelson C. C. Davis R. A. (2015). Cytotoxic C20 Diterpenoid Alkaloids from the Australian Endemic Rainforest Plant Anopterus macleayanus. J. Nat. Prod 78, 2908–2916. 10.1021/acs.jnatprod.5b00509 26600001

[B25] Li C. M. Liu Z. L. Mu Q. Sun H. D. Zheng H. L. Tao G. D. (1997). Studies on chemical constituents from leaves of Goniothalamus Griffithii. Actabot. yunnanica 19, 321–323.

[B26] Li C. M. Mu Q. Sun H. D. Xu B. Tang W. D. Zheng H. L. (1998). A new anti-cancer constituent of Goniothalamuscheliensis. Actabot. yunnanica 20, 102–104.

[B27] Li X. An J. Li H. Qiu X. Wei Y. Shang Y. (2019). The methyl-triclosan induced caspase-dependent mitochondrial apoptosis in HepG2 cells mediated through oxidative stress. Ecotoxicol Environ. Saf. 182, 109391. 10.1016/j.ecoenv.2019.109391 31272020

[B28] Lim H. Kim S. Y. Lee E. Lee S. Oh S. Jung J. (2019). Sex-Dependent Adverse Drug Reactions to 5-Fluorouracil in Colorectal Cancer. Biol. Pharm. Bull. 42, 594–600. 10.1248/bpb.b18-00707 30930418

[B29] Liou J. R. Wu T. Y. Thang T. D. Hwang T. L. Wu C. C. Cheng Y. B. (2014). Bioactive 6S-styryllactone constituents of Polyalthia parviflora. J. Nat. Prod 77, 2626–2632. 10.1021/np5004577 25419616

[B30] Luo Y. Wang X. Wang H. Xu Y. Wen Q. Fan S. (2015). High Bak Expression Is Associated with a Favorable Prognosis in Breast Cancer and Sensitizes Breast Cancer Cells to Paclitaxel. PloS One 10, e0138955. 10.1145/2818302 26406239 PMC4583467

[B34] Mu Q. Li C. M. Zhang H. J. Sun H. D. (1996). A styryllactone from Goniothalamus leiocapus. Chin. Chem. Lett. 7, 617–618.

[B33] Mu Q. Li C. M. Sun H. D. Zheng H. L. Tao G. D. (1998). The Chemical constituents of Goniothalamus leiocarpus. Actabot. yunnanica 20, 123–125.

[B32] Mu Q. Li C. M. He Y. N. Sun H. D. Zheng H. L. Lu Y. (1999a). A new styryl-lactone compound from Goniothalamus leiocarpus. Chin. Chem. Lett. 10, 135–138.

[B35] Mu Q. Tang W. D. Li C. M. Lu Y. Sun H. D. Zheng H. L. (1999b). Four new Styryllactone from Goniothalamus leiocarpus. Heterocycles 51, 2969–2976. 10.3987/COM-99-8679

[B36] Mu Q. Tang W. D. Li C. M. Xu Y. P. Sun H. D. Lou L. G. (2002). The absolute configuration of Leiocarpin, B. Heterocycles 57, 337–340. 10.3987/COM-01-9399

[B37] Mu Q. Tang W. D. Liu R. Y. Li C. M. Lou L. G. Sun H. D. (2003). Constituents from the stems of Goniothalamus griffithii. Planta Med. 69, 826–830. 10.1055/s-2003-43219 14598208

[B31] Mu Q. He Y. N. Tang W. D. Li C. M. Lou L. G. Sun H. D. (2004). A styrylpyrone dimer from Bark of Goniothalamus leiocarpus. Chin. Chem. Lett. 15, 191–193.

[B38] Peeters J. Neeskens P. Tollenaere J. P. Van Remoortere P. Brewster M. E. (2002). Characterization of the interaction of 2-hydroxypropyl-beta-cyclodextrin with itraconazole at pH 2, 4, and 7. J. Pharm. Sci. 91, 1414–1422. 10.1002/jps.10126 12115841

[B39] Peris E. Estornell E. Cabedo N. Cortes D. Bermejo A. (2000). 3-acetylaltholactone and related styryl-lactones, mitochondrial respiratory chain inhibitors. Phytochemistry 54, 311–315. 10.1016/S0031-9422(00)00104-7 10870186

[B40] Pohl M. Schmiegel W. (2016). Therapeutic Strategies in Diseases of the Digestive Tract - 2015 and Beyond Targeted Therapies in Colon Cancer Today and Tomorrow. Dig Dis. 34, 574–579. 10.1159/000445267 27332557

[B41] Purushotham A. D. Lewison G. Sullivan R. (2012). The state of research and development in global cancer surgery. Ann. Surg. 255, 427–432. 10.1097/SLA.0b013e318246591f 22281701

[B42] Ruden M. Puri N. (2013). Novel anticancer therapeutics targeting telomerase. Cancer Treat Rev. 39, 444–456. 10.1016/j.ctrv.2012.06.007 22841437

[B43] Semprebon S. C. De Fatima A. Lepri S. R. Sartori D. Ribeiro L. R. Mantovani M. S. (2014). (S)-Goniothalamin induces DNA damage, apoptosis, and decrease in BIRC5 messenger RNA levels in NCI-H460 cells. Hum. Exp. Toxicol. 33, 3–13. 10.1177/0960327113491506 23749456

[B44] Siegel R. Desantis C. Jemal A. (2014). Colorectal cancer statistics 2014. CA Cancer J. Clin. 64, 104–117. 10.3322/caac.21220 24639052

[B45] Sridhar R. Ravanan S. Venugopal J. R. Sundarrajan S. Pliszka D. Sivasubramanian S. (2014). Curcumin- and natural extract-loaded nanofibres for potential treatment of lung and breast cancer: in vitro efficacy evaluation. J. Biomater Sci. Polym Ed 25, 985–998. 10.1080/09205063.2014.917039 24865590

[B46] Sunkara V. Hebert J. R. (2015). The colorectal cancer mortality-to-incidence ratio as an indicator of global cancer screening and care. Cancer 121, 1563–1569. 10.1002/cncr.29228 25572676 PMC4424055

[B47] Trieu Q. H. Mai H. D. Pham V. C. Litaudon M. Gueritte F. Retailleau P. (2014). Styryllactones and acetogenins from the fruits of Goniothalamus macrocalyx. Nat. Prod Commun. 9, 495–498. 10.1177/1934578X1400900417 24868866

[B48] Vemuri S. K. Banala R. R. Mukherjee S. Uppula P. Gpv S. A. V. G. (2019). Novel biosynthesized gold nanoparticles as anti-cancer agents against breast cancer: Synthesis, biological evaluation, molecular modelling studies. Mater Sci. Eng C Mater Biol. Appl. 99, 417–429. 10.1016/j.msec.2019.01.123 30889716

[B49] Vendramini-Costa D. B. Alcaide A. Pelizzaro-Rocha K. J. Talero E. Avila-Roman J. Garcia-Maurino S. (2016). Goniothalamin prevents the development of chemically induced and spontaneous colitis in rodents and induces apoptosis in the HT-29 human colon tumor cell line. Toxicol. Appl. Pharmacol. 300, 1–12. 10.1016/j.taap.2016.03.009 27016270

[B51] Wang F. Yang B. Zhao Y. Liao X. Gao C. Jiang R. (2014a). Host-guest inclusion system of scutellarein with 2-hydroxypropyl-beta-cyclodextrin: preparation, characterization, and anticancer activity. J. Biomater Sci. Polym Ed 25, 594–607. 10.1080/09205063.2014.884875 24555409

[B53] Wang Z. X. Cao J. X. Liu Z. P. Cui Y. X. Li C. Y. Li D. (2014b). Combination of chemotherapy and immunotherapy for colon cancer in China: a meta-analysis. World J. Gastroenterol. 20, 1095–1106. 10.3748/wjg.v20.i4.1095 24574784 PMC3921535

[B52] Wang Y. Wang J. Wang H. Ye W. (2016). Novel taxane derivatives from Taxus wallichiana with high anticancer potency on tumor cells. Chem. Biol. Drug Des. 88, 556–561. 10.1111/cbdd.12782 27153813

[B50] Wang A. Wang M. Pang Q. Jia L. Zhao J. Chen M. (2018). Lily extracts inhibit the proliferation of gastric carcinoma SGC-7901 cells by affecting cell cycle progression and apoptosis via the upregulation of caspase-3 and Fas proteins, and the downregulation of FasL protein. Oncol. Lett. 16, 1397–1404. 10.3892/ol.2018.8806 30008816 PMC6036323

[B54] Yen H. K. Fauzi A. R. Din L. B. Mckelvey-Martin V. J. Meng C. K. Inayat-Hussain S. H. (2014). Involvement of Seladin-1 in goniothalamin-induced apoptosis in urinary bladder cancer cells. BMC Complement Altern. Med. 14, 295. 10.1186/1472-6882-14-295 25107315 PMC4150971

[B55] Zhang J. Gao G. Chen L. Li J. Deng X. Zhao Q. S. (2014). Hydrogen peroxide/ATR-Chk2 activation mediates p53 protein stabilization and anti-cancer activity of cheliensisin A in human cancer cells. Oncotarget 5, 841–852. 10.18632/oncotarget.1780 24553354 PMC3996661

[B56] Zhao D. Gong T. Fu Y. Nie Y. He L. L. Liu J. (2008). Lyophilized Cheliensisin A submicron emulsion for intravenous injection: characterization, in vitro and in vivo antitumor effect. Int. J. Pharm. 357, 139–147. 10.1016/j.ijpharm.2008.01.055 18329194

