# Supplementary material for: Hydroxypropyl-β-Cyclodextrin Complexes of Styryllactones Enhance the Anti-Tumor Effect in SW1116 Cell Line
Source: Front Pharmacol. 2020 Apr 22;11:484. doi: 10.3389/fphar.2020.00484 (PMC7188779; doi:10.3389/fphar.2020.00484)
Supplement: Supplementary file 1 [file DataSheet_1.pdf]

Supplementary Material

1 Supplementary Figures and Tables

1.1 Supplementary Tables

TABLE 1. Crystal data for cheliensisin A

Empirical formula	$C_{15}H_{14}O_5$
Formula weight	274.26
Temperature	123 (2) K
Wavelength	1.54178 Å
θ range for data collection	4.72 - 67.48°
Crystal system	Orthorhombic
Space group	P212121
	a = 7.0297 (10) Å, α= 90°
Unit cell dimensions	b = 11.0918(10) Å, β= 90°
	c = 17.5287 (3) Å, γ = 90°
Volume	1366.75 (3) $Å^3$
Z	4
Calculated density	1.333 Mg/m^3
Absorption coefficient	0.842 mm^{-1}
F(000)	576
Crystal size	0.21×0.15×0.14 mm^3
Index ranges	-8 ≤ h ≤ 8, -13≤ k ≤ 13, -18≤ l ≤ 20
Reflections collected	8080
Independent reflections	2435 [*R*(int) = 0.0300]
Completeness to theta=65.96°	99.9%
Absorption correction	Semi-empirical from equivalents
Max and min transmission	0.7456 and 0.3475
Refinement method	Full-matrix least-squares on F^2
Data/restraints/parameters	2435/0/183
Goodness-of-fit on F^2	1.068
Final R indices[I>2sigma(I)]	R1=0.0272, wR2= 0.0670
R indices (all data)	R1=0.0274, wR2 = 0.0673

TABLE 2. Crystal data for leiocarpin B

Empirical formula	$C_{28}H_{24}O_7$
Formula weight	472.47
Temperature	140 (2) K
Wavelength	1.54178 Å
θ range for data collection	3.15 – 69.15°
Crystal system	Monoclinic
Space group	C2
	a = 21.0501 (4) Å, α= 90°,
Unit cell dimensions	b = 7.9013 (10) Å, β= 121.2160 (10)°
	c = 16.4209 (3) Å, γ = 90°
Volume	2335.75 (7) $Å^3$
Z	4
Calculated density	1.344 Mg/m^3
Absorption coefficient	0.798 mm^{-1}
F(000)	992
Crystal size	0.35×0.26×0.20 mm^3
Index ranges	$-25 \leq h \leq 25$, $-9\leq k \leq 9$, $-19 \leq l \leq 19$
Reflections collected	6301
Independent reflections	3373[R(int) = 0.0432]
Completeness to theta=65.96°	98.2%
Absorption correction	semi-empirical from equivalents;
Max and min transmission	0.7532 and 0.4386
Refinement method	full-matrix least-squares on F^2
Data/restraints/parameters	3373/1/319
Goodness-of-fit on F^2	1.079
Final R indices[I>2sigma(I)]	R1=0.0419, wR2= 0.1082
R indices (all data)	R1= 0.0421, wR2 = 0.1089

TABLE 3. Crystal data for leiocarpin E

Empirical formula	$C_{26}H_{24}O_6$
Formula weight	432.45
Temperature	123 (2) K
Wavelength	1.54178 Å
θ range for data collection	4.34 - 65.50°
Crystal system	Orthorhombic
Space group	P212121
	a = 9.7380 (10) Å, α= 90°
Unit cell dimensions	b = 11.1611 (2) Å, β= 90°
	c = 20.3823 (3) Å, γ = 90°
Volume	2215.29 (6) $Å^3$
Z	4
Calculated density	1.297 Mg/m^3
Absorption coefficient	0.754 mm^{-1}
F(000)	912
Crystal size	0.19×0.15×0.12 mm^3
Index ranges	$-11 \leq h \leq 11$, $-13\leq k \leq 13$, $-24 \leq l \leq 24$
Reflections collected	12242
Independent reflections	3746[R(int) = 0.0432]
Completeness to theta=65.96°	99.5%
Absorption correction	semi-empirical from equivalents
Max and min transmission	0.9129 and 0.8706;
Refinement method	full-matrix least-squares on F^2
Data/restraints/parameters	3746/0/291
Goodness-of-fit on F^2	1.058
Final R indices[I>2sigma(I)]	R1=0.0426, wR2 = 0.1191
R indices (all data)	R1= 0.0430, wR2 = 0.1195

TABLE 4. Crystal data for goniodiol

Empirical formula	$C_{13}H_{14}O_4$
Formula weight	234.24
Temperature	T = 140(2) K
Wavelength	1.54178 Å
θ range for data collection	5.66–69.43°
Crystal system	Orthorhombic
Space group	P212121
Unit cell dimensions	a = 9.2443 (2) Å, α = 90°<br>b = 9.7650 (2) Å, β = 90°,<br>c = 13.0267 (3) Å, γ = 90°
Volume	1175.93 (4) $Å^3$
Z	4
Calculated density	1.317 Mg/m^3
Absorption coefficient	0.814 mm^{-1}
F(000)	492
Crystal size	0.35× 0.26× 0.22 mm^3
Index ranges	-10≤ h ≤ 10, -10≤ k ≤ 11, -14≤ l≤ 15;
Reflections collected	5322
Independent reflections	2070[R(int) = 0.0369]
Completeness to theta=65.96°	98.1%
Absorption correction	semi-empirical from equivalents;
Max and min transmission	0.7532 and 0.5891
Refinement method	full-matrix least-squares on F^2
Data/restraints/parameters	2070/0/157
Goodness-of-fit on F^2	1.118
Final R indices[I>2sigma(I)]	R1=0.0417, wR2= 0.1044
R indices (all data)	R1= 0.0418, wR2=0.1045

TABLE 5. Crystal data for goniodiol-7-monoacetate

Empirical formula	$C_{15}H_{16}O_5$
Formula weight	276.28
Temperature	T = 296(2) K
Wavelength	1.54178 Å
θ range for data collection	2.90–69.66°;
Crystal system	Triclinic
Space group	P1
	a = 5.4547 (5) Å, α = 94.379(6)°
Unit cell dimensions	b = 8.8394 (7) Å, β = 91.949(5)°
	c = 15.3120 (13) Å, γ = 105.106(6)°
Volume	709.58 (11) $Å^3$
Z	2
Calculated density	1.293 Mg/m^3
Absorption coefficient	0.811 mm^{-1}
F(000)	292
Crystal size	0.2× 0.12× 0.05 mm^3
Index ranges	$-6 \leq h \leq 6$, $-10 \leq k \leq 10$, $-18 \leq l \leq 15$
Reflections collected	5692
Independent reflections	3307[R(int) = 0.0419]
Completeness to theta=65.96°	94.0%
Absorption correction	semi-empirical from equivalents
Max and min transmission	0.7532 and 0.4727;
Refinement method	full-matrix least-squares on F^2
Data/restraints/parameters	3307/3/365
Goodness-of-fit on F^2	1.042
Final R indices[I>2sigma(I)]	R1=0.0468, wR2= 0.1251
R indices (all data)	R1= 0.0505, wR2=0.1309
